# Sterile Inflammatory Synovitis as a Mimic for Prosthetic Joint Infection in Patients With Rheumatoid Arthritis Following Total Knee Arthroplasty: A Report of Two Cases

**DOI:** 10.7759/cureus.78766

**Published:** 2025-02-09

**Authors:** Conor Jones, Anne Debenedetti, Craig Della Valle

**Affiliations:** 1 Orthopaedic Surgery, Rush University Medical Center, Chicago, USA

**Keywords:** post-operative complication, prosthetic joint infection, rheumatoid arthritis, sterile inflammatory synovitis, total knee arthroplasty

## Abstract

Patients with rheumatoid arthritis (RA) present with unique challenges following total knee arthroplasty (TKA). Rarely, these patients may present with sterile inflammatory synovitis with a clinical picture that can mimic prosthetic joint infection (PJI).

We report on two patients with RA who underwent primary TKA performed by the senior author who presented with sterile inflammatory synovitis following TKA. Both patients presented several years after their index procedure with knee pain and effusion, concerning for PJI. Both patients underwent extensive evaluations for infection. Initial aspirations showed a synovial fluid white blood cell count of more than 3,000 WBC/uL but a differential of less than 80% and no growth on the final culture. Both patients were treated non-operatively without antibiotics with the resolution of their symptoms.

Sterile inflammatory synovitis is a rare post-operative complication that can present among patients with RA following TKA. While the initial presentation and evaluation may be concerning for PJI, a thorough laboratory evaluation must be performed to accurately diagnose these patients. The use of next-generation DNA sequencing, Synovasure alpha-defensin, and Synovasure microbial ID panel can aid in diagnoses. These patients may be treated without antibiotics or operative intervention. A thorough evaluation for PJI should be performed to ensure the correct diagnosis and appropriate management.

## Introduction

Rheumatoid arthritis (RA) is an autoimmune condition characterized by synovitis of multiple joints with eventual cartilage erosion and destruction [1]. Although RA may be treated medically with non-steroidal anti-inflammatory drugs, courses of steroids, and disease-modifying antirheumatic drugs, many patients face debilitating end-stage arthritis, eventually requiring arthroplasty [2,3]. As both the life expectancy of patients with RA and the volume of primary total knee arthroplasty (TKA) continue to increase, coupled with advances in surgical techniques and implant design, a greater number of patients with RA are undergoing TKA [4,5]. Compared to patients with conventional osteoarthritis (OA), patients undergoing TKA for RA are at increased risk for multiple post-operative complications, including prosthetic joint infection (PJI) [6-8].

RA has been identified as a strong risk factor for PJI, with studies suggesting that PJI is a major cause of failure among patients with RA and that the evaluation of these patients should be similar to those with non-inflammatory arthropathy [9-11]. Cipriano et al. reviewed 61 patients with inflammatory arthritis following total joint arthroplasty and identified optimal cut-offs of 3450 cells/mL synovial fluid white blood cells (WBCs), 78% synovial polymorphonuclear leukocytes (PMNs), 32 mm/h erythrocyte sedimentation rate (ESR), and 15 mg/L C-reactive protein (CRP), suggesting cut-off values that are similar to patients without inflammatory arthritis [11].

Despite this, we have encountered two patients with RA who we believe presented with a sterile synovitis after TKA. We initially were concerned about PJI in both cases; however, after an extensive evaluation and with careful monitoring, both patients were treated non-operatively and without antibiotics with the complete resolution of their symptoms. While PJI should be at the top of the differential diagnosis list for RA patients who present with the onset of pain and swelling post-TKA, an awareness of this rare entity could prevent unnecessary re-operations if this condition is identified.

## Case presentation

Case 1

A 68-year-old female patient with a history of RA underwent a right TKA in December 2019 without complications. In May of 2023, she presented to our clinic with intermittent right knee pain and effusions. An examination of her knee revealed a large effusion. 100 cc of cloudy synovial fluid was aspirated from her right knee and sent for cell count, differential, and cultures. The cell count and differential resulted in 9,271 WBCs and 46% PMNs, respectively. The patient's synovial WBC count was concerning for PJI as the number of WBCs exceeded the threshold of 3,000 WBCs for PJI. However, the percentage of PMNs was below 80%, which is the threshold of a PJI. Aerobic and anaerobic cultures from that aspirate resulted in no growth.

Given the inconclusive nature of the first aspiration, a repeat aspiration was performed two weeks later including alpha-defensin and next-generation DNA sequencing, a second culture for aerobic and anaerobic organisms, as well as a culture for fungal and acid-fast bacilli. The cell count of the repeat aspiration was 10,810 WBCs with 40.8% PMNs. The next-generation DNA sequencing was negative, and Synovasure alpha-defensin was negative. Synovasure microbial ID panel, which detects microbial antigens within the synovial fluid, was negative. Aerobic, anaerobic, fungal, and acid-fast bacilli cultures all finalized in no growth. After a thorough clinical and laboratory work-up, the patient's presentation was inconsistent with PJI. She was given a corticosteroid injection and nine months following her corticosteroid injection reports that her knee symptoms and effusions have resolved.

Case 2

A 79-year-old female patient with a history of RA underwent a left TKA in April 2019. She presented in August 2023 with atraumatic, intermittent left knee pain. She endorsed general tenderness over her left knee as well as recurrent effusions. On examination, her left knee was noted to have a moderate effusion and generalized tenderness. The left knee was aspirated and showed a synovial fluid WBC count of 15,325 and 65% PMNs. Aerobic and anaerobic cultures from that aspirate resulted in no growth.

The left knee aspiration was repeated one week later which showed a synovial fluid WBC of 2,202 cells/uL and 16.1% PMNs. Next-generation DNA sequencing was negative, Synovasure alpha-defensin was negative, and Synovasure microbial ID panel identified no fungal or bacterial antigens. Repeat cultures were all negative. These results were inconsistent with PJI. Antibiotic therapy and surgical intervention were deferred. Six months following this episode, she has no symptoms, and her effusions have resolved.

Table 1 shows the synovial fluid analysis of RA patients following TKA.

**Table 1:** Synovial fluid analysis of RA patients following TKA RA: rheumatoid arthritis; TKA: total knee arthroplasty; WBC: white blood cells; PMNs: polymorphonuclear leukocytes

	Case 1	Case 2	Reference range
First aspiration			
Synovial fluid WBC (cells/uL)	9,271	15,325	<3,000
Synovial differential (% PMNs)	46%	65%	<80%
Crystal analysis	None present	None present	None present
Aerobic culture	No growth	No growth	No growth
Anaerobic culture	No growth	No growth	No growth
Fungal culture	No growth	No growth	No growth
Acid-fast bacilli culture	No growth	No growth	No growth
Second aspiration			
Synovial fluid WBC (cells/uL)	10,810	2,202	<3,000
Synovial differential (% PMNs)	40.8%	16.1%	<80%
Crystal analysis	None present	None present	None present
Aerobic culture	No growth	No growth	No growth
Anaerobic culture	No growth	No growth	No growth
Fungal culture	No growth	No growth	No growth
Acid-fast bacilli culture	No growth	No growth	No growth
Next-generation DNA sequencing bacteria	Negative	Negative	Negative
Next-generation DNA sequencing fungi	Negative	Negative	Negative
Synovasure alpha-defensin	Negative	Negative	Negative
Synovasure microbial ID panel			
*Propionibacterium acnes *panel	Negative	Negative	Negative
*Staphylococcus *panel	Negative	Negative	Negative
*Candida *panel	Negative	Negative	Negative
*Enterococcus *panel	Negative	Negative	Negative

## Discussion

RA is a chronic disease characterized by the overproduction of tumor necrosis factor (TNF), leading to synovial inflammation and joint destruction [1]. Patients with RA will often have recurrent effusions secondary to synovial inflammation in their native knee. Although medical advancements within the past 30 years have mitigated the impact of this disease process, many patients with RA still require TKA for the treatment of their knee arthritis [4,5]. The purpose of this case report is to inform other providers of the risk for recurrent sterile inflammatory synovitis in RA patients following TKA, which may mimic PJI on initial treatment. Correctly diagnosing sterile inflammatory synovitis may prevent unnecessary surgical interventions and antibiotic prescriptions as the treatment for PJI commonly involves surgical irrigation and debridement with implant exchange and a prolonged course of antibiotics.

Post-operative flares following TKA have been described previously in the literature [12]. Patients with RA are asked to discontinue biologics prior to surgery, and a known precipitant of RA flares is medication withdrawal. These flares can present with painful effusions. However, post-operative RA flares have only been described in the immediate post-operative period. Our cases describe patients who were several years after their surgery, and both were on their home RA regimen.

Next-generation DNA sequencing has been found to be a useful adjunct in the work-up of potential PJI. This tool allows for sequencing all DNA in a sample to provide a more complete picture of the microbial profile present. Previous work by Tarabichi et al. demonstrated that next-generation sequencing was able to identify an organism in 82% of cases of PJI that were previously culture-negative [13]. In both of our cases, next-generation DNA sequencing was negative. In addition to next-generation sequencing, Synovasure alpha-defensin was used as a part of the work-up for PJI. Alpha-defensin is a peptide secreted by neutrophils and has been found to be highly sensitive for PJI [14,15]. Although neutrophils were present in both aspirates, alpha-defensin was negative in both instances, which aided us in ruling out infection.

In both instances, repeat aspirations were used as a part of a comprehensive laboratory evaluation. Hassebrock et al. evaluated the role of repeat joint aspirations in patients with concern for PJI following total hip arthroplasty [16]. They reported that repeat aspiration changed the diagnosis of PJI in 43.3% of cases and deemed repeat aspirations a useful tool in patients with conflicting clinical data. 

In patients with RA, management of inflammatory synovitis during TKA procedures has been an area of focus [5]. Prior work has suggested the use of a complete synovectomy to reduce the risk of recurrent synovitis, while other groups have advocated that a partial synovectomy may be appropriate in cases of mild RA [5,17,18]. In both of our cases, each patient underwent a complete synovectomy during their index procedure, yet each developed sterile inflammatory synovitis. Although the role of synovectomy in patients with RA undergoing TKA is ongoing, these cases suggest that patients with RA are at risk of recurrent sterile inflammatory synovitis following TKA that can mirror PJI even after a complete synovectomy.

## Conclusions

Patients with RA may rarely present with sterile inflammatory synovitis following TKA with a clinical picture and aspirate concerning for PJI. A thorough evaluation for PJI should be performed to ensure the correct diagnosis and appropriate management. These patients may be treated with anti-inflammatories alone with the eventual resolution of their symptoms, avoiding unnecessary return trips to the operating room and antibiotics. Physicians should be aware of this clinical diagnosis when evaluating patients with RA for possible PJI.
